# Interaction Between First-Trimester Energy-Adjusted Dietary Inflammatory Index and Educational Level on the Risk of Anemia During the Second and Third Trimesters: A Prospective Cohort Study

**DOI:** 10.3390/nu17203241

**Published:** 2025-10-15

**Authors:** Fan Xia, Cong Huang, Zhitan Zhang, Junwei He, Hongzhuan Tan, Tingting Wang, Lizhang Chen, Mengshi Chen, Jing Deng

**Affiliations:** 1 Department of Epidemiology and Health Statistics, Xiangya School of Public Health, Central South University, No. 172 Tongzipo Road, Yuelu District, Changsha 410013, China; 226901005@csu.edu.cn (F.X.); huangcc2000@163.com (C.H.); 18661893668@163.com (Z.Z.); hjw001007@163.com (J.H.); tanhz@mail.csu.edu.cn (H.T.); wangting91123@126.com (T.W.); liche4005@126.com (L.C.); mancechen@foxmail.com (M.C.); 2 Hunan Provincial Key Laboratory of Clinical Epidemiology, Xiangya School of Public Health, Central South University, No. 172 Tongzipo Road, Yuelu District, Changsha 410013, China

**Keywords:** energy-adjusted dietary inflammatory index, pro-inflammatory diet, anemia, educational level, pregnancy

## Abstract

**Objectives**: The aim of this cohort study was to assess the associations of first-trimester energy-adjusted Dietary Inflammatory Index (E-DII) and maternal educational level with anemia during the second and third trimesters, as well as their potential interactions. **Methods**: This study enrolled 562 eligible pregnant women. Multivariable modified Poisson regression with robust variance was used to assess the associations of first-trimester E-DII and maternal educational level with anemia during the second and third trimesters. Restricted cubic splines (RCS) explored nonlinear associations, while additive and multiplicative interaction models evaluated the interaction between first-trimester E-DII and maternal education. **Results**: The participants, with a mean age of 29.45 ± 4.28 years, had an anemia incidence of 14.59% during the second and third trimesters. In fully adjusted models, higher first-trimester E-DII (T3 vs. T1) significantly increased anemia risk (RR = 2.30, 95% CI: 1.36–3.90). Lower education (below bachelor’s degree) independently elevated anemia risk (RR = 2.27, 95% CI: 1.52–3.39). RCS revealed no significant nonlinear relationship between the E-DII and anemia (*p* > 0.05). Although no significant multiplicative interaction was observed, a positive additive interaction was identified between first-trimester E-DII and educational level on the risk of anemia after adjustment for covariates, including age, ethnicity, pre-pregnancy BMI, employment, and baseline serum iron, among others. The measures of additive interaction were statistically significant: RERI = 4.64 (95% CI: 1.51–11.34), AP = 0.68 (95% CI: 0.26–0.86), and S = 4.91 (95% CI: 1.16–20.69) (all *p* < 0.05). **Conclusions**: First-trimester pro-inflammatory diets and lower educational attainment independently predicted anemia during the second and third trimesters and demonstrated a significant positive additive interaction. Combined nutritional and educational interventions integrated into prenatal care targeting pregnant women with limited education could effectively reduce anemia in pregnancy and improve perinatal outcomes.

## 1. Introduction

Anemia is a highly prevalent complication of pregnancy with substantial consequences for maternal and infant health globally. Recent global estimates (as of 2023) indicate that approximately 36% of pregnant women are affected by anemia, primarily driven by iron deficiency, which accounts for about 40% of cases [1,2]. Mirroring this global pattern, a 2024 meta-analysis of 57 studies on Chinese pregnant women reported a pooled anemia prevalence of 30.7% [3], with a significantly higher occurrence during the second and third trimesters than in early pregnancy [3,4]. Mid- and late-pregnancy anemia may compromise placental function and impair nutrient and oxygen supply to the fetus, thereby increasing the risk of low birth weight [5]. Maternal iron deficiency has been associated with long-term cognitive deficits in offspring, with males potentially facing higher risks during early developmental stages [6]. Additionally, anemia in the third trimester is linked to an elevated incidence of preterm birth [7] and gestational hypertension [8]. Therefore, reducing the prevalence of anemia in mid- and late pregnancy is of critical importance for improving maternal and neonatal health outcomes.

Anemia in pregnancy is closely associated with deficiencies in multiple micronutrients, with iron, folate, and vitamin B12 playing pivotal roles. Iron deficiency impairs hemoglobin (Hb) synthesis, leading to microcytic hypochromic anemia [9]. As a critical coenzyme in one-carbon metabolism, folate is essential for DNA synthesis. Deficiency in folate disrupts erythroid precursor proliferation and results in megaloblastic anemia [9]. Vitamin B12, which supports DNA synthesis and erythrocyte maturation, similarly contributes to megaloblastic changes and ineffective erythropoiesis when inadequate [10]. Current clinical guidelines widely recommend routine iron and folic acid supplementation for pregnant women [9]. Moreover, cohort studies have revealed that vitamin B12 and folate interact in early pregnancy to modulate hematological parameters such as mean corpuscular volume (MCV) [11,12], suggesting that combined supplementation with iron, folate, and vitamin B12 may significantly reduce the risk of anemia.

The pathogenesis of anemia in pregnancy extends beyond nutritional deficiencies to encompass physiological hemodilution, chronic blood loss from conditions such as helminthiasis, and inherited hemoglobin disorders, with growing evidence also underscoring the role of inflammation as a key contributor [13,14]. In particular, inflammatory processes may mediate iron-refractory anemia by impairing iron utilization, even in the presence of adequate iron stores [13]. Diet is a key modulator of such inflammatory processes, as evidenced by research showing that dietary patterns significantly influence systemic inflammation. The Dietary Inflammatory Index (DII) provides a quantitative tool to assess this relationship, scoring individual diets based on 45 components according to their inflammatory impact [15]. A lower DII score reflects an anti-inflammatory dietary pattern, whereas a higher score indicates a pro-inflammatory diet [16]. The Energy-Adjusted Dietary Inflammatory Index (E-DII) further refines this approach by calculating inflammatory potential per 1000 kilocalories consumed, thereby accounting for energy intake and reducing confounding by caloric consumption [17,18]. This adjustment is particularly useful in early pregnancy, where nausea and vomiting often cause substantial variation in energy intake. By controlling for total calories, the E-DII offers a more accurate and robust measure of dietary inflammatory patterns during the first-trimester. Biologically, pro-inflammatory diets can elevate systemic inflammation, increasing cytokines such as IL-6 that stimulate hepcidin production, leading to iron sequestration and reduced availability for erythropoiesis [19,20]. This mechanism suggests that first-trimester dietary inflammation may establish a persistent dysregulation of iron metabolism, thereby contributing to anemia risk in later pregnancy. Nevertheless, evidence regarding the influence of dietary inflammation on gestational anemia remains limited.

In addition to dietary patterns and supplement use, socioeconomic factors, especially maternal educational attainment, have also been associated with the risk of anemia in pregnancy [21]. As a readily obtainable demographic characteristic, maternal education level can be efficiently utilized to identify high-risk populations for targeted screening and timely interventions, thereby highlighting the practical significance of investigating its association with anemia in pregnancy. Evidence from an Indian study demonstrated a positive association between higher maternal education levels and improved Hb concentrations during pregnancy [22]. This relationship is potentially mediated through the effect of education on health literacy, which promotes the acquisition of nutritional knowledge required for selecting iron-rich foods and appropriate prenatal supplements [23,24,25]. Furthermore, lower educational levels are frequently linked to socioeconomic status that shape dietary patterns, often leading to higher consumption of pro-inflammatory processed foods and reduced intake of micronutrient-dense options [26,27,28]. Such diets are reflected in elevated E-DII scores, which may further exacerbate the risk of anemia. Given that both lower education and higher E-DII are independent risk factors for anemia in pregnancy, exploring their potential interaction could facilitate the identification of high-risk subgroups and provide a crucial theoretical basis for the prevention of gestational anemia.

In summary, anemia during the second and third trimesters is associated with adverse effects on both maternal and fetal health. Beyond well-established nutritional risk factors, including deficiencies in iron and folic acid, pro-inflammatory dietary patterns and lower maternal education may represent significant contributors to anemia development. While the E-DII provides a more accurate assessment of dietary inflammatory patterns in early pregnancy by accounting for energy intake variations due to nausea and vomiting, prospective evidence linking first-trimester E-DII to anemia in later pregnancy remains limited. This prospective cohort study investigates the association between first-trimester E-DII and the risk of anemia in mid- and late gestation, as well as assesses the potential interaction between maternal educational attainment and E-DII. Given that educational level represents a readily measurable demographic variable, elucidating its potential interactive role with pro-inflammatory diets could enhance the identification of high-risk subgroups. The findings could guide the design of targeted nutritional interventions and educational strategies, potentially improving resource allocation and reducing the burden of anemia in pregnancy.

## 2. Materials and Methods

### 2.1. Study Design and Participants

We utilized a prospective cohort established at a maternal and child health hospital in Hunan Province, China (2017–2019). Specifically, pregnant women were recruited according to the following criteria:

(1) Singleton pregnancy;

(2) Regular follow-up at the study hospital;

(3) Capacity to provide informed consent with signed documentation.

Exclusion criteria comprised:

(1) Missing Hb data in any trimester;

(2) Anemia development during first-trimester;

(3) Acute gestational infections (to eliminate confounding effects [29]), including febrile respiratory illness, symptomatic urinary tract infection, clinically diagnosed viral infection, or chorioamnionitis. These cases were excluded because such infections trigger systemic inflammation and hepcidin-mediated iron sequestration, which can transiently alter iron and hemoglobin status independently of diet [13,19,29,30];

(4) Missing baseline serum iron and key questionnaire data (diet, education).

**Figure 1:**
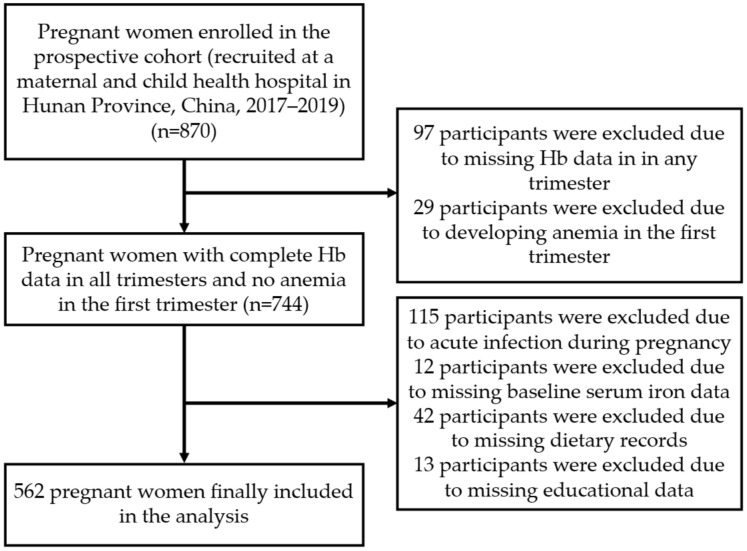
STROBE flow diagram of participant selection.

Of the 870 women initially enrolled, 562 progressed to the final analysis following the screening and exclusion process detailed in Figure 1.

Anemia was diagnosed according to the World Health Organization (WHO) criteria using gestational age-specific Hb cutoffs: <110 g/L for the first and third trimesters, and <105 g/L for the second trimester [31]. The primary outcome was a composite of anemia incidence, defined as the development of anemia at either the second or third trimester, with each participant counted only once. Hb concentration was measured in venous whole blood using an automated hematology analyzer with standard colorimetric techniques. Blood sampling for Hb measurement was scheduled during the second (24–27 weeks) and third (28 weeks until delivery) trimesters.

The study protocol received ethical approval (No. EC201624) from the Institutional Ethics Committee of Hunan Provincial Maternal and Child Health Hospital, with written informed consent provided by all participants following a detailed explanation of the study and prior to enrollment.

### 2.2. Sample Size Calculation

This prospective cohort study was designed to evaluate the association between the first-trimester E-DII and the risk of anemia during the second and third trimesters. A post hoc sample size calculation was performed based on the observed data from the study, which showed an anemia incidence of 14.59% in the reference group and a relative risk (RR) of 2.30 associated with the E-DII. Using a two-sided α of 0.05 and β of 0.1 (90% power), and applying the standard formula for cohort studies, the minimum required total sample size was calculated to be 210 participants. The final cohort comprised 562 participants, which substantially exceeds this requirement and ensures a high statistical power of over 99.9% for the observed effect, thus affirming the robustness of the findings.

(1) $$N=\frac{{\left(Z_{1-\alpha /2}\sqrt{2pq}+Z_{\beta }\sqrt{p_{0}q_{0}+p_{1}q_{1}}\right)}^{2}}{{\left(p_{1}-p_{0}\right)}^{2}}$$

### 2.3. Data Collection

Data collection was conducted during the first trimester (weeks 11–13) by a team of four uniformly trained researchers using a comprehensive questionnaire. The instrument comprised three modules: a general survey, a risk factor assessment, and a food frequency questionnaire (FFQ). All researchers held bachelor’s degrees or higher in public health or clinical medicine and had completed standardized study-specific training. To ensure data quality, all questionnaires underwent cross-checking by different team members to identify omissions and logical inconsistencies.

(1) The general survey captured demographic and obstetric characteristics, including age, ethnicity, pre-pregnancy body mass index (BMI), educational level, employment status, household income, health insurance coverage, parity, gravidity and history of adverse pregnancy outcomes;

(2) The survey questionnaire and physical examination records provided baseline clinical and lifestyle data: baseline serum iron levels; use of iron, folic acid, and vitamin B12 supplements; vomiting, menstrual blood loss, and physical activity; and smoking and alcohol history, secondhand smoke exposure, Pittsburgh Sleep Quality Index (PSQI) score [32,33], and Edinburgh Postnatal Depression Scale (EPDS) score [34,35].

(3) Dietary intake over the preceding month was assessed using a validated FFQ [36], which categorized foods into thirteen groups: cereals, tubers, fruits, vegetables, poultry and livestock meats, freshwater products, seafood, dairy products, soybean products, nuts, edible oils, beverages and alcoholic beverages. Frequency options ranged from “never” to “daily.” Participants self-reported portion sizes. Nutrient supplement use was documented through an inventory that captured both physician-recommended and self-prescribed supplements, the latter referring to those voluntarily chosen and purchased by participants without a physician’s directive. Nutrient intake from these self-prescribed supplements was quantified based on the standardized nutritional labeling provided by the manufacturers. These data on dietary intake and supplement use were subsequently used to calculate the E-DII, as detailed in the following section.

### 2.4. Calculation of E-DII

The DII, developed by Shivappa et al., quantifies the inflammatory potential of an individual’s diet based on 45 dietary parameters, each assigned an inflammatory effect score derived from global nutritional data across 11 populations [16]. The DII remains valid even when fewer than 30 nutrient components are included in its calculation [37,38]. In this study, the DII was computed using 26 out of the 45 parameters: carbohydrates; protein; total fat; monounsaturated fatty acids (MUFAs); polyunsaturated fatty acids (PUFAs); saturated fat; *n*-3 and *n*-6 fatty acids; cholesterol; fiber; vitamins B1, B2, B6, B12, A, C, D, and E; folate; niacin; β-carotene; magnesium; iron; zinc; selenium; and energy. A complete mapping of the 26 dietary parameters used in this study against the original 45 DII parameters is provided in Supplementary Table S1. For each dietary component, a Z-score was calculated by subtracting the global mean intake from the participant’s reported intake and dividing the result by the global standard deviation. Each Z-score was subsequently converted to a percentile value, which was then centered by doubling and subtracting one. The resulting value was multiplied by the respective food parameter-specific inflammatory effect score. Finally, the overall DII score for each participant was derived by summing the products of all dietary components. To account for variations in energy intake, an E-DII was calculated by expressing dietary components per 1000 kcal and repeating the standardization and summation process [39]. For the E-DII, 25 parameters were used, as total energy was used as the denominator for energy adjustment.

Although the original DII was designed to assess the inflammatory potential of diet alone, we calculated the E-DII both with and without supplements for several key reasons. First, dietary supplements provide concentrated doses of nutrients that can significantly modulate systemic inflammation [40]. Second, excluding them might introduce residual confounding, as supplement use is associated with both inflammation levels and anemia in pregnancy [9,10,40]. Furthermore, this comprehensive approach aligns with the methodology established by the DII developers and applied in prior nutritional epidemiology studies [41]. The DII, employed here including supplements, was used in our prior research on pregnancy complications [42]. Consequently, we created two variables: the primary exposure was the E-DII including supplements (hereafter referred to as ‘E-DII scores’), representing the overall inflammatory load from total nutrient intake; the E-DII excluding supplements was also calculated to assess the contribution of the habitual diet alone. Both were utilized to thoroughly examine their associations with anemia in the second and third trimesters.

Among the 562 participants who completed the dietary assessment, the association between E-DII scores and anemia during the second and third trimesters was assessed using tertile categorizations (T1: ≤−0.68; T2: −0.68~0.81; T3: ≥0.81) [43,44,45]. Similarly, the E-DII scores excluding supplements were also divided into tertiles (T1: ≤−0.64; T2: −0.64~0.87; T3: ≥0.87).

For the evaluation of interaction between E-DII and education level, the E-DII was necessarily dichotomized as the calculation of additive interaction measures requires both exposures to be binary. We employed the median value of 0.07 as the cutoff, categorizing participants into a biologically meaningful ‘anti-inflammatory diet’ group (E-DII ≤ 0.07) and a ‘pro-inflammatory diet’ group (E-DII > 0.07). This approach for dichotomizing the E-DII has been used in prior studies [46,47], providing a rationale for its application here to define anti- and pro-inflammatory dietary patterns in our cohort. To ensure robustness, sensitivity analyses employing alternative binary categorizations (T3 vs. T1; T2 vs. T1; and T3 vs. T1 + T2) were also conducted, as detailed in the Supplementary Material (Tables S2 and S3).

### 2.5. Variable Definition

(1) Age: Maternal age was categorized into two groups consisting of under 35 years and 35 years or older, with the latter classified as advanced maternal age [48].
(2) Pre-pregnancy BMI: It was classified as underweight (<18.5 kg/m^2^), normal weight (18.5–24.0 kg/m^2^), or overweight/obesity (≥24.0 kg/m^2^) [49].
(3) Pre-pregnancy regular physical activity: Exercise more than three times a week for more than 30 min each time.
(4) Sleep quality: It was assessed using the PSQI, a 19-item instrument evaluating seven sleep components. The global PSQI score ranges from 0 to 21, with total scores categorized as very good (≤5), good (6–10), or poor (≥11) [32].
(5) Depression: Depressive symptoms were assessed using the EPDS, which comprises 10 items rated on a 4-point scale. Total scores range from 0 to 30, with a score ≤ 12 indicating no clinically significant symptoms and a score ≥ 13 indicating clinically significant depressive symptoms [50].
(6) First-trimester E-DII: This refers to the E-DII calculated from both dietary and supplemental nutrient intakes. It was the primary exposure variable in this study, representing the overall inflammatory potential of total nutrient intake. It was employed as a continuous variable, in tertiles (T1–T3) for association analyses, and as a binary variable for interaction analysis.
(7) First-trimester E-DII excluding supplements: This was a supplementary exposure variable, calculated from dietary sources only. It was used to assess the inflammatory potential inherent to the habitual diet, independent of supplement use, and was also analyzed in tertiles (T1–T3).

### 2.6. Statistical Analysis

Statistical analyses were conducted using SPSS 26.0 and R 4.5.0. A two-sided α level of 0.05 was used to define statistical significance. Quantitative variables are summarized as mean ± standard deviation (if normally distributed) or median with interquartile range (IQR) (if non-normally distributed), and categorical variables are expressed as frequencies and percentages. Group comparisons were performed using independent samples *t*-tests for normally distributed quantitative variables, Mann–Whitney U tests for non-normally distributed quantitative variables, and chi-square tests for categorical variables.

Given that the composite outcome of anemia during the second and third trimesters was not rare, with an incidence of 14.59%, odds ratios (OR) derived from logistic regression could overestimate the strength of associations compared to risk ratios (RR). Therefore, the associations between E-DII, educational level, and anemia were primarily assessed using modified Poisson regression with robust standard errors to directly estimate interpretable RRs. For comparison, ORs from multivariable logistic regression were also calculated but are presented exclusively in the Supplementary Materials. To avoid over-adjustment, the use of iron, folic acid, or vitamin B12 supplements was not included as covariates in these primary multivariable models, as their intake is already captured within the E-DII calculation. Additionally, in R 4.5.0, the potential nonlinear relationship between first-trimester E-DII and anemia during the second and third trimesters was assessed by incorporating restricted cubic splines (RCS) using the splines package into the modified Poisson model with robust variance (estimated with the sandwich and lmtest packages). Nonlinearity was tested using a likelihood ratio test from the car package, with results visualized using ggplot2. Furthermore, to investigate the specific influence of including nutrient supplements in the E-DII calculation, we conducted sensitivity analyses using a first-trimester E-DII score that excluded all supplements. The association of this modified exposure with anemia was assessed using modified Poisson regression in the entire cohort and in subgroups of non-users of iron or vitamin B12 supplements, with all models appropriately adjusted for supplement use.

The additive and multiplicative interactions between first-trimester E-DII and educational level in the risk of anemia were evaluated using the interactionR package in R 4.5.0. While E-DII was analyzed as tertiles in previous associations, it was dichotomized for interaction analyses because the assessment of additive interaction requires both exposures to be binary. Using the median value of 0.07 as the cutoff, participants were categorized into anti-inflammatory (E-DII ≤ 0.07) and pro-inflammatory (E-DII > 0.07) diet groups, consistent with prior literature [46,47]. Educational level was similarly categorized as below bachelor’s degree versus bachelor’s degree and above. The reference category for all interaction measures was the group with an anti-inflammatory diet (E-DII ≤ 0.07) and higher education (bachelor’s degree or above). Additive interaction was evaluated using three metrics: the relative excess risk due to interaction (RERI), the attributable proportion due to interaction (AP), and the synergy index (S). A positive additive interaction was considered present if RERI and its 95% confidence interval (CI) were greater than 0, AP and its 95% CI were greater than 0, and S and its 95% CI were greater than 1. Multiplicative interaction was assessed by including a product term between E-DII and education level. A positive multiplicative interaction was considered present if the multiplicative scale and its 95% CI were greater than 1. Sensitivity analyses employing alternative binary categorizations of E-DII (T3 vs. T1; T2 vs. T1; and T3 vs. T1 + T2) were also conducted.

## 3. Results

### 3.1. General Characteristics of Participants

A total of 562 pregnant women were included in this study. Overall, the mean maternal age was 29.45 ± 4.28 years, while the mean pre-pregnancy BMI was 20.75 ± 2.65 kg/m^2^. Anemia was identified in 22 women during the second trimester, of which 7 cases were moderate and 15 were mild. In the third trimester, 60 women were diagnosed with anemia, all of which were mild. The incidence of anemia during the second and third trimesters was 14.59%.

As shown in Table 1, the distribution of educational attainment differed significantly between groups (*p* < 0.001), with a lower proportion of women holding a bachelor’s degree or above in the anemia group (53.66%) compared to the non-anemia group (73.54%). In terms of dietary inflammatory patterns, the median first-trimester E-DII score for the cohort was 0.07 (IQR: −1.10 to 1.23). The first-trimester E-DII score was significantly higher in the anemia group (median: 0.74) than in the non-anemia group (median: 0.00) (*p* < 0.001), indicating a more pro-inflammatory diet among women who subsequently developed anemia. Furthermore, a significantly higher proportion of women in the anemia group were in the highest E-DII tertile (T3, E-DII ≥ 0.81) compared to the non-anemia group (48.78% vs. 30.62%, *p* < 0.01). In contrast, the first-trimester E-DII excluding supplements (median for the cohort: 0.10; IQR: −1.07 to 1.10) showed no significant association with anemia during the second and third trimesters, with no differences between groups in either the continuous score or the tertile distribution (all *p* > 0.05).

### 3.2. Relationship Between First-Trimester E-DII and Anemia During the Second and Third Trimesters

The association between first-trimester E-DII and anemia risk in the second and third trimesters was evaluated using modified Poisson regression. As shown in Table 2, a higher pro-inflammatory E-DII score was consistently associated with an increased risk of anemia. In the fully adjusted model, each one-unit increase in the continuous E-DII score was associated with a 27% increase in the risk of anemia (RR = 1.27, 95% CI: 1.13–1.42). Women in the highest E-DII tertile (T3) had 2.30 times the risk of anemia compared to those in the lowest tertile (T1) (RR = 2.30, 95% CI: 1.36–3.90). For comparison, ORs from multivariable logistic regression are provided in Supplementary Table S4. As anticipated given the non-rare outcome incidence (14.59%), the ORs demonstrated an overestimation of the effect size compared to the RRs (e.g., OR = 1.34 vs. RR = 1.27 for the continuous score in the fully adjusted model).

**Figure 2:**
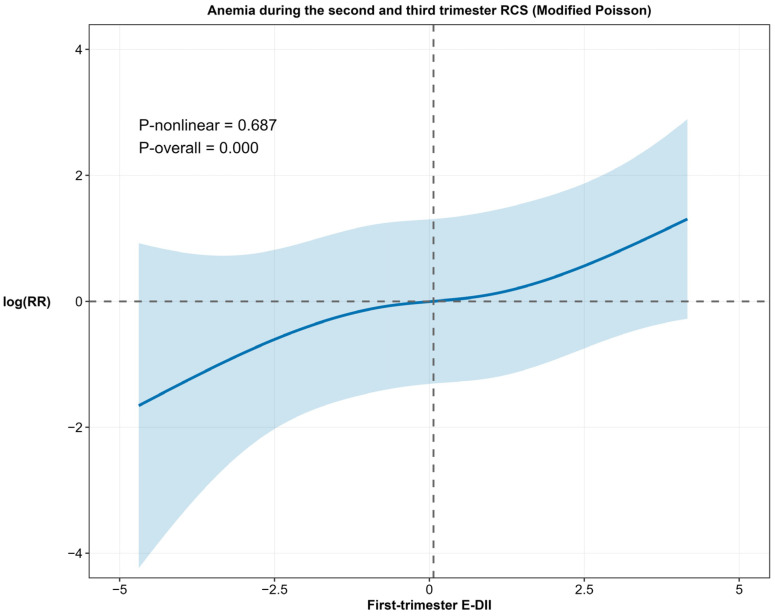
Restricted cubic spline (RCS) regression of the association between the first-trimester E-DII and anemia during the second and third trimesters. The model was based on a modified Poisson regression with robust variance and adjusted for age, ethnicity, pre-pregnancy BMI, educational level, employment, monthly household income, health insurance status, history of adverse pregnancy outcomes, gravidity, parity, baseline serum iron, and vomiting. First-trimester E-DII refers to the E-DII calculated from both dietary and supplemental nutrient intakes, thereby obviating the need for separate adjustment of individual supplement use. The solid blue line represents the estimated log(RR), and the blue shaded area indicates the 95% CI.

The nature of this association was further delineated using RCS analysis. As shown in Figure 2, the RCS plot, based on the adjusted modified Poisson model, revealed a significant linear dose–response relationship between the first-trimester E-DII score and anemia risk (*p* for overall association < 0.001; *p* for nonlinearity = 0.687), visually confirming the steady increase in risk with rising E-DII scores.

Finally, we investigated the influence of nutrient supplements by analyzing a first-trimester E-DII score calculated without supplements. As summarized in Supplementary Table S5, after comprehensive adjustment for multiple covariates including supplement use, no significant associations were observed in the full cohort or among non-iron or non-vitamin B12 supplement users. Specifically, in the fully adjusted model for the entire population, the continuous E-DII score excluding supplements showed no significant association with anemia risk (RR = 1.12, 95% CI: 0.98–1.28, *p* = 0.096), with no clear trend across tertiles.

### 3.3. Relationship Between Educational Level and Anemia During the Second and Third Trimesters

Multivariable modified Poisson regression was employed to evaluate associations between maternal educational level and anemia during the second and third trimesters (Table 3). A significant association was observed between lower education and anemia risk in the unadjusted model (*p* < 0.001). It remained significant and was slightly strengthened in the fully adjusted model (Model 2) after controlling for demographic factors, clinical factors, and first-trimester E-DII, with women with an educational level below bachelor’s degree having 2.27 times the risk of anemia (RR = 2.27, 95% CI: 1.52–3.39). This association demonstrates that lower educational attainment constitutes an independent risk factor for anemia during the second and third trimesters. For comparison, corresponding ORs from logistic regression are provided in Supplementary Table S6.

### 3.4. The Interaction Between First-Trimester E-DII and Educational Level on Anemia During the Second and Third Trimesters

Additive and multiplicative interaction models were employed to evaluate the interaction between first-trimester E-DII and educational level on anemia during the second and third trimesters. As detailed in Table 4, the multiplicative interaction was not statistically significant (*p* > 0.05). In contrast, after multivariable adjustment for covariates including age, ethnicity, pre-pregnancy BMI, employment, monthly household income, health insurance status, history of adverse pregnancy outcomes, gravidity, parity, baseline serum iron, and vomiting, the analysis revealed a statistically significant positive additive interaction (RERI = 4.64, 95% CI: 1.51–11.34; AP = 0.68, 95% CI: 0.26–0.86; S = 4.91, 95% CI: 1.16–20.69). Women with both a pro-inflammatory diet and lower educational attainment had the highest risk, with a 6.83-fold increased risk of anemia compared to those with an anti-inflammatory diet and higher education (*p* < 0.001). This positive additive interaction identifies women with both a first-trimester pro-inflammatory diet and lower education as a high-risk population for anemia in mid-to-late pregnancy.

To assess whether these findings were sensitive to the choice of cutoff, we conducted sensitivity analyses using alternative binary categorizations of E-DII (T3 vs. T1; T2 vs. T1; and T3 vs. T1 + T2). As detailed in Supplementary Tables S2 and S3, these analyses did not yield statistically significant additive or multiplicative interactions. This lack of significance in the sensitivity analyses likely reflects reduced statistical power when comparing smaller subgroups (each comprising approximately one-third of the cohort), in contrast to the median split approach used in our primary analysis that maximizes sample size in each exposure category.

## 4. Discussion

This prospective cohort study identified a 14.59% prevalence of anemia during the second and third trimesters. After adjusting for potential confounders, both a pro-inflammatory diet in the first-trimester (assessed by E-DII including supplements) and lower maternal educational attainment were independently associated with an increased anemia risk, with a 2.30-fold elevated risk observed for pro-inflammatory diets. Furthermore, a significant positive additive interaction between lower education and pro-inflammatory diet was demonstrated, highlighting their synergistic effect on anemia development and supporting the identification of high-risk subgroups for targeted interventions.

Although direct evidence regarding the association between the E-DII and anemia in pregnancy remains limited, this study demonstrated that elevated first-trimester E-DII scores were significantly associated with an increased risk of anemia in both mid- and late pregnancy, a finding biologically plausible and consistent with mechanisms linking systemic inflammation to impaired iron metabolism. Higher E-DII scores have been associated with elevated systemic inflammatory markers, including IL-6, CRP, and TNF-α [51,52], which contribute to hepcidin upregulation [19,20], with IL-6 in particular enhancing hepcidin expression via the JAK/STAT3 pathway [53]. Increased hepcidin expression subsequently induces the internalization and degradation of ferroportin, the principal cellular iron exporter, thereby inhibiting iron efflux from enterocytes, hepatocytes, and macrophages [19,54,55]. This cascade leads to sequestered iron storage, reduced circulating iron availability, and ultimately may contribute to the development of anemia, particularly in the context of elevated dietary inflammation during pregnancy. In this study, potential confounding from acute inflammation was mitigated by excluding women with diagnosed gestational infections. Nevertheless, the absence of baseline CRP and ferritin meant that residual confounding by subclinical inflammation or iron stores remained a consideration for the observed associations. In contrast, a previous investigation reported no significant association between first-trimester DII and anemia in the third trimester [56]. This discrepancy may be attributed to the fact that nausea and vomiting of pregnancy substantially disrupt total dietary intake, thereby distorting the original DII, which is based on absolute intake levels. In this study, the E-DII effectively reduced this bias through energy intake standardization, thereby enabling a more accurate representation of the intrinsic inflammatory quality of dietary patterns and enhancing the validity of inflammatory potential assessment. Our finding that a pro-inflammatory diet in early pregnancy increases subsequent anemia risk is consistent with a report from a Japanese cohort, which observed an association between a higher E-DII in the third trimester and lower hemoglobin levels [57].

A critical finding was that the significant association with anemia was specific to the E-DII that included dietary supplements, while the diet-alone E-DII showed no association. This discrepancy suggested that anti-inflammatory nutrients contained in supplements played a critical role in modulating the overall inflammatory burden. Evidence indicated that supplemental intake of vitamin E, particularly in combination with other micronutrients such as vitamin C, B vitamins (B1, B2, B6, B12, folic acid, niacin), and selenium, was associated with significantly lower concentrations of CRP, a key inflammatory marker [40]. Although iron, folic acid, and vitamin B12 were directly involved in hematopoiesis, their supplemental forms might also have mitigated inflammation. The absence of a significant association for the E-DII excluding supplements, particularly in the subgroups of non-iron and non-vitamin B12 supplement users, further supported this interpretation. This lack of association may be attributed to the diet-alone E-DII’s failure to capture the impact of other anti-inflammatory supplements (e.g., vitamins E and C) commonly used in these populations, though limited statistical power in these subgroups could also have contributed to the null results. Consequently, the E-DII including supplements provided a more biologically comprehensive estimate of the net inflammatory load, whereas reliance solely on dietary sources likely led to exposure misclassification by omitting the substantive anti-inflammatory contributions of supplemental nutrients.

It was observed that lower maternal educational attainment was significantly associated with an elevated risk of anemia during the second and third trimesters, after comprehensive adjustment for demographic, socioeconomic, behavioral factors, and the first-trimester E-DII. This finding was supported by population-based studies from China [58], in which an educational level of ≤9 years was consistently associated with a higher prevalence of anemia. Notably, in Brazil, a 54% increased anemia in pregnancy risk was observed among women with ≤4 years of education compared to those with more than 12 years of education [59]. The observed association in our analysis could be explained by several interrelated pathways. Lower educational attainment was often associated with reduced health literacy, which could adversely affect nutritional knowledge, overall dietary quality [26,60], and the ability to access and utilize prenatal health information. Furthermore, women with less education were more likely to adopt pro-inflammatory dietary patterns and have inadequate micronutrient intake [61], which could disrupt iron metabolism, especially during the high-demand late stages of pregnancy. Although we adjusted for key socioeconomic confounders (income, health insurance) and dietary inflammatory potential, residual confounding by unmeasured factors such as detailed health literacy, healthcare-seeking behaviors, and other aspects of diet quality cannot be entirely ruled out. Based on these findings, it was recommended that simplified nutritional counseling be integrated into routine antenatal care for pregnant women with lower educational backgrounds. Additionally, incorporating anemia prevention into broader public health education and community-based initiatives, particularly in regions with marked educational disparities [58], was advised to mitigate this modifiable risk factor.

In this study, we revealed a positive additive interaction between the maternal education level below bachelor’s degree and higher first-trimester E-DII in elevating the risk of anemia during the second and third trimesters. Unlike previous studies that primarily attributed anemia risk solely to the effect of DII [56,57], our study identified maternal education as an important effect modifier, revealing that socioeconomic disparities can amplify the impact of dietary inflammation—a dimension that has not been previously described in the literature. The RERI of 4.64 indicated that the excess risk of anemia associated with the combined exposure to both factors exceeded the sum of the excess risks associated with each factor alone by 4.64 units of relative risk. Consequently, targeted interventions for this high-risk subgroup are anticipated to yield a disproportionate benefit in reducing the population-level burden of anemia. This synergy was further confirmed by an AP of 0.68 (95% CI: 0.26–0.86), suggesting that 68% of the anemia risk in this high-risk group was specifically attributable to the interaction itself. The absence of a significant multiplicative interaction highlighted that this synergistic relationship was best captured on the additive scale, emphasizing its relevance for public health intervention planning. These findings underscore the necessity for integrated interventions addressing both socioeconomic and nutritional factors. Given that the observed associations were specific to the E-DII that included dietary supplements, nutritional counseling should explicitly emphasize the role of anti-inflammatory supplements alongside dietary improvements. For women with lower educational attainment, interventions could focus on practical guidance regarding supplement selection and use. For their higher-educated counterparts, strategies should aim to reinforce existing knowledge about maintaining anti-inflammatory practices. Such a targeted approach would effectively address the specific risk profile revealed by our findings and provide an efficient framework for reducing the anemia burden in pregnancy.

## 5. Limitations

This study has several limitations. First, the assessment of iron status and underlying inflammation was constrained by the lack of ferritin and high-sensitivity CRP data, relying instead on serum iron. Our study lacked biochemical measurements of vitamin B12 and folic acid levels as these specific biomarkers were not included in the original data collection protocol of the pre-existing cohort. Second, the generalizability of our findings may be limited, as participants were recruited from a single province and the cohort included a high proportion of highly educated women. Third, although necessary to control for acute inflammatory confounders, the exclusion of women with gestational infections and those lost to follow-up may have introduced selection bias. Finally, dietary data were self-reported and thus subject to measurement error and potential social desirability bias, with the reporting of portion sizes and intake frequencies potentially affecting the accuracy of the E-DII calculations.

## 6. Conclusions

It was demonstrated that pro-inflammatory dietary patterns in the first trimester and lower maternal education significantly increased the risk of anemia during the second and third trimesters, with a positive additive interaction further amplifying this risk. From a public health perspective, these findings underscore the necessity of implementing integrated nutritional and educational interventions, particularly for pregnant women with lower education levels. Future antenatal care should incorporate personalized anti-inflammatory dietary guidance that emphasizes the appropriate use of nutritional supplements. Such an approach would effectively reduce the burden of gestational anemia and improve maternal and neonatal outcomes.

## Figures and Tables

**Table 1:** General characteristics of subgroups of pregnant women with anemia during the second and third trimesters.

Characteristics	Second- and Third-Trimester Non-Anemia Group (*n* = 480)	Second- and Third-Trimester Anemia Group (*n* = 82)	Overall (*n* = 562)	Statistical Value	*p* Value
Age (years)				0.006	0.938
<35	420 (87.50)	72 (87.80)	492 (87.54)		
≥35	60 (12.50)	10 (12.20)	70 (12.46)		
Ethnicity				1.400	0.237
Han ethnic group	457 (95.21)	81 (98.78)	538 (95.73)		
Other	23 (4.79)	1 (1.22)	24 (4.27)		
Pre-pregnancy BMI (kg/m^2^)	20.84 ± 2.70	20.26 ± 2.25	20.75 ± 2.65	1.822	0.069
Pre-pregnancy BMI group				4.846	0.089
<18.5	93 (19.38)	16 (19.51)	109 (19.40)		
18.5–24.0	323 (67.29)	62 (75.61)	385 (68.50)		
≥24.0	64 (13.33)	4 (4.88)	68 (12.10)		
Educational level				13.350	<0.001 ***
Bachelor’s degree and above	353 (73.54)	44 (53.66)	397 (70.64)		
Below bachelor’s degree	127 (26.46)	38 (46.34)	165 (29.36)		
Employment				0.006	0.938
Unemployed	60 (12.50)	10 (12.20)	70 (12.46)		
Employed	420 (87.50)	72 (87.80)	492 (87.54)		
Monthly household income (RMB)				1.469	0.226
≤10,000	276 (57.50)	53 (64.63)	329 (58.54)		
>10,000	204 (42.50)	29 (35.37)	233 (41.46)		
Health insurance status				0.067	0.796
Uninsured	33 (6.88)	5 (6.10)	38 (6.76)		
Insured	447 (93.12)	77 (93.90)	524 (93.24)		
History of adverse pregnancy outcomes				1.152	0.283
No	280 (58.33)	53 (64.63)	333 (59.25)		
Yes	200 (41.67)	29 (35.37)	229 (40.75)		
Gravidity				0.146	0.929
1	206 (42.92)	37 (45.12)	243 (43.24)		
2	142 (29.58)	23 (28.05)	165 (29.36)		
≥3	132 (27.50)	22 (26.83)	154 (27.40)		
Parity				0.007	0.934
0	295 (61.46)	50 (60.98)	345 (61.39)		
≥1	185 (38.54)	32 (39.02)	217 (38.61)		
Baseline serum iron (mmol/L)	8.75 ± 0.85	8.69 ± 0.82	8.74 ± 0.85	0.573	0.567
Iron supplementation				2.016	0.156
Yes	204 (42.5)	28 (34.15)	232 (41.28)		
No	276 (57.5)	54 (65.85)	330 (58.72)		
Folic acid supplementation				3.011	0.083
Yes	452 (94.17)	73 (89.02)	525 (93.42)		
No	28 (5.83)	9 (10.98)	37 (6.58)		
Vitamin B12 supplementation				2.943	0.086
Yes	182 (37.92)	23 (28.05)	205 (36.48)		
No	298 (62.08)	59 (71.95)	357 (63.52)		
Vomiting(days)				2.570	0.109
<30	245 (51.04)	34 (41.46)	279 (49.64)		
≥30	235 (48.96)	48 (58.54)	283 (50.36)		
Menstrual blood loss (ml)				1.867	0.393
<30	32 (6.67)	3 (3.66)	35 (6.23)		
30–80	434 (90.42)	75 (91.46)	509 (90.57)		
>80	14 (2.91)	4 (4.88)	18 (3.2)		
Pre-pregnancy regular physical activity				0.389	0.533
No	347 (72.29)	62 (75.61)	409 (72.78)		
Yes	133 (27.71)	20 (24.39)	153 (27.22)		
Pre-pregnancy smoking history				0.008	0.928
No	470 (97.92)	81 (98.78)	551 (98.04)		
Yes	10 (2.08)	1 (1.22)	11 (1.96)		
Pre-pregnancy alcohol consumption				<0.001	1.000
No	468 (97.50)	80 (97.56)	548 (97.51)		
Yes	12 (2.50)	2 (2.44)	14 (2.49)		
Secondhand smoke exposure				2.196	0.138
No	382 (79.58)	71 (86.59)	453 (80.6)		
Yes	98 (20.42)	11 (13.41)	109 (19.4)		
Sleep quality (PSQI score classification)				0.442	0.802
Very good	97 (20.21)	15 (18.29)	112 (19.93)		
Good	275 (57.29)	46 (56.10)	321 (57.12)		
Poor	108 (22.50)	21 (25.61)	129 (22.95)		
Depression (EPDS score classification)				0.015	0.901
No	418 (87.08)	71 (86.59)	489 (87.01)		
Yes	62 (12.92)	11 (13.41)	73 (12.99)		
First-trimester E-DII score	0.00 (2.36)	0.74 (2.49)	0.07 (2.33)	24,452	<0.001 ***
First-trimester E-DII (groups)				11.806	0.003 **
T1 (≤−0.68)	171 (35.63)	17 (20.73)	188 (33.46)		
T2 (−0.68~0.81)	162 (33.75)	25 (30.49)	187 (33.27)		
T3 (≥ 0.81)	147 (30.62)	40 (48.78)	187 (33.27)		
First-trimester E-DII excluding supplements	0.06 (2.16)	0.26 (2.22)	0.10 (2.17)	21473	0.187
First-trimester E-DII excluding supplements (groups)				1.281	0.527
T1 (≤−0.64)	165 (34.38)	23 (28.05)	188 (33.46)		
T2 (−0.64~0.87)	158 (32.92)	29 (35.37)	187 (33.27)		
T3 (≥0.87)	157 (32.70)	30 (36.58)	187 (33.27)		

Note: **: *p* < 0.01; ***: *p* < 0.001. First-trimester E-DII refers to the E-DII calculated from both dietary and supplemental nutrient intakes.

**Table 2:** Relationship between first-trimester E-DII and anemia during the second and third trimesters (RR and 95% CI).

Variable	Model 1		Model 2	
	RR (95%CI)	*p* Value	RR (95%CI)	*p* Value
First-trimester E-DII score	1.25 (1.11, 1.41)	<0.001 ***	1.27 (1.13, 1.42)	<0.001 ***
First-trimester E-DII (groups)				
T1 (≤−0.68)	1 (Ref.)	-	1 (Ref.)	-
T2 (−0.68~0.81)	1.48 (0.83, 2.65)	0.188	1.45 (0.80, 2.66)	0.223
T3 (≥0.81)	2.37 (1.39, 4.02)	0.001 **	2.30 (1.36, 3.90)	0.002 **

Note: Model 1 represents the basic model without adjustment for covariates. Model 2 was adjusted for the age, ethnicity, pre-pregnancy BMI, educational level, employment, monthly household income, health insurance status, history of adverse pregnancy outcomes, gravidity, parity, baseline serum iron and vomiting. First-trimester E-DII refers to the E-DII calculated from both dietary and supplemental nutrient intakes, thereby obviating the need for separate adjustment of individual supplement use. **: *p* < 0.01; ***: *p* < 0.001.

**Table 3:** Relationship between educational level and anemia during the second and third trimesters (RR and 95% CI).

Variable	Model 1		Model 2	
	RR (95%CI)	*p* Value	RR (95%CI)	*p* Value
Educational level				
Bachelor’s degree and above	1 (Ref.)	-	1 (Ref.)	-
Below bachelor’s degree	2.08 (1.40, 3.08)	<0.001 ***	2.27 (1.52, 3.39)	<0.001 ***

Note: Model 1 represents the basic model without adjustment for covariates. Model 2 was adjusted for the age, ethnicity, pre-pregnancy BMI, employment, monthly household income, health insurance status, history of adverse pregnancy outcomes, gravidity, parity, baseline serum iron, vomiting and first-trimester E-DII (groups). First-trimester E-DII refers to the E-DII calculated from both dietary and supplemental nutrient intakes, thereby obviating the need for separate adjustment of individual supplement use. ***: *p* < 0.001.

**Table 4:** Additive and multiplicative interaction between first-trimester E-DII and educational level on the risk of anemia during the second and third trimesters.

Variable	Educational Level			RERI ^a^	AP ^a^	SI ^a^	Multiplicative Scale ^a^
	Bachelor’s Degree and Above	Below Bachelor’s Degree	Effect of Below Bachelor’s Degree Within the Strata of E-DII				
First-trimester E-DII (groups)				4.64 (1.51, 11.34) *	0.68 (0.26, 0.86) ***	4.91 (1.16, 20.69) *	2.69 (0.93, 7.78)
anti-inflammatory (≤0.07)	1 (Ref.)	1.63 (0.70, 3.80)	1.63 (0.70, 3.80)				
pro-inflammatory (>0.07)	1.56 (0.81, 3.00)	6.83 (3.27, 14.26) ***	4.39 (2.24, 8.61) ***				
Effect of pro-inflammatory within the strata of educational level	1.56 (0.81, 3.00)	4.19 (1.83, 9.56) ***					

Note: ^a^ The RERI, AP, SI and Multiplicative scale was adjusted for the age, ethnicity, pre-pregnancy BMI, employment, monthly household income, health insurance status, history of adverse pregnancy outcomes, gravidity, parity, baseline serum iron and vomiting. First-trimester E-DII refers to the E-DII calculated from both dietary and supplemental nutrient intakes, thereby obviating the need for separate adjustment of individual supplement use. *: *p* < 0.05; ***: *p* < 0.001.

## Data Availability

The data that support the findings of this study are available from the corresponding author upon reasonable request. The data are not publicly available due to privacy restrictions and confidentiality agreements.

## Supplementary Materials

The following supporting information can be downloaded at: https://www.mdpi.com/article/10.3390/nu17203241/s1, Table S1: Selection and application of original DII parameters for DII and E-DII calculation; Table S2: Sensitivity analysis of the E-DII and educational level interaction on anemia risk using alternative binary categorizations of E-DII (T3 vs. T1 and T2 vs. T1); Table S3: Sensitivity analysis of the E-DII and educational level interaction on anemia risk using alternative binary categorizations of E-DII (T3 vs. T1+T2); Table S4: Association between first-trimester E-DII and anemia during the second and third trimesters (OR with 95% CI from logistic regression and RR with 95% CI from modified Poisson regression); Table S5: Association between first-trimester E-DII excluding supplements and anemia during the second and third trimesters, in the overall population and in specific subgroups of non-supplement users (RR and 95% CI); Table S6: Association between educational level and anemia during the second and third trimesters (OR with 95% CI from logistic regression and RR with 95% CI from modified Poisson regression).
