# Supplementary material for: Interaction Between First-Trimester Energy-Adjusted Dietary Inflammatory Index and Educational Level on the Risk of Anemia During the Second and Third Trimesters: A Prospective Cohort Study
Source: Nutrients. 2025 Oct 15;17(20):3241. doi: 10.3390/nu17203241 (PMC12566625; doi:10.3390/nu17203241)
Supplement: Supplementary file 1 [file nutrients-17-03241-s001.zip › nutrients-3897999-supplementary.pdf]

Supplementary Table S1. Selection and application of original DII parameters for DII and E-DII calculation.

Original DII Food Parameter	Included in DII Calculation in this Study	Included in E-DII Calculation in this Study	Overall Inflammatory Effect Score
Energy (kcal)	Yes	No	0.180
Carbohydrate (g)	Yes	Yes	0.097
Protein (g)	Yes	Yes	0.021
Total fat (g)	Yes	Yes	0.298
Saturated fat (g)	Yes	Yes	0.373
MUFA (g)	Yes	Yes	-0.009
PUFA (g)	Yes	Yes	-0.337
n-3 Fatty acids (g)	Yes	Yes	-0.436
n-6 Fatty acids (g)	Yes	Yes	-0.159
Cholesterol (mg)	Yes	Yes	0.110
Fibre (g)	Yes	Yes	-0.663
Thiamin (mg)	Yes	Yes	-0.098
Riboflavin (mg)	Yes	Yes	-0.068
Niacin (mg)	Yes	Yes	-0.246
Vitamin B6 (mg)	Yes	Yes	-0.365
Vitamin B12 (μg)	Yes	Yes	0.106
Folic acid (μg)	Yes	Yes	-0.190
Vitamin A (RE)	Yes	Yes	-0.401
Vitamin C (mg)	Yes	Yes	-0.424
Vitamin D (μg)	Yes	Yes	-0.446
Vitamin E (mg)	Yes	Yes	-0.419
β-Carotene (μg)	Yes	Yes	-0.584
Mg (mg)	Yes	Yes	-0.484
Fe (mg)	Yes	Yes	0.032
Zn (mg)	Yes	Yes	-0.313
Se (μg)	Yes	Yes	-0.191
Alcohol (g)	No	No	-0.278
Caffeine (g)	No	No	-0.110
Eugenol (mg)	No	No	-0.140
Garlic (g)	No	No	-0.412
Ginger (g)	No	No	-0.453
Onion (g)	No	No	-0.301
Saffron (g)	No	No	-0.140
Trans fat (g)	No	No	0.229
Turmeric (mg)	No	No	-0.785
Green/black tea (g)	No	No	-0.536
Flavan-3-ol (mg)	No	No	-0.415
Flavones (mg)	No	No	-0.616

Original DII Food Parameter	Included in DII Calculation in this Study	Included in E-DII Calculation in this Study	Overall Inflammatory Effect Score
Flavonols (mg)	No	No	-0.467
Flavonones (mg)	No	No	-0.250
Anthocyanidins (mg)	No	No	-0.131
Isoflavones (mg)	No	No	-0.593
Pepper (g)	No	No	-0.131
Thyme/oregano (mg)	No	No	-0.102
Rosemary (mg)	No	No	-0.013

Note: The table lists all 45 original parameters. The 26 parameters used for the overall DII calculation in this study are listed first, followed by the 19 not utilized. For theE-DII, the 'Energy' parameter was excluded from standardization (as it served as the denominator for energy adjustment), resulting in 25 parameters used in the E-DII calculation. The inflammatory effect scores are derived from the original DII development cohort.

Supplementary Table S2. Sensitivity analysis of the E-DII and educational level interaction on anemia risk using alternative binary categorizations of E-DII (T3 vs. T1 and T2 vs. T1).

Variable	Educational level			RERI [a]	AP [a]	SI [a]	Multiplicative scale [a]
	Bachelor's degree and above	Below bachelor's degree	Effect of below bachelor's degree within the strata of E-DII				
First-trimester E-DII（groups)							
T1 (≤ -0.68)	1(Ref.)	3.10 (1.06, 9.04)*	3.10 (1.06, 9.04)*				
T2 (-0.68~0.81)	1.69 (0.68, 4.20)	4.40 (1.61, 12.05)**	2.60 (1.01, 6.68)*	0.88 (-5.24, 7.52)	0.20 (-1.41, 0.68)	1.35 (0.32, 5.63)	0.92 (0.22, 3.85)
T3 (≥ 0.81)	2.88 (1.24,6.66)*	7.27 (2.88,18.39)***	2.53 (1.12, 5.70)*	2.30 (-4.62,11.81)	0.32 (-0.72,0.67)	1.58 (0.55,4.54)	0.82 (0.21,3.10)
Effect of T2 within the strata of educational level	1.69 (0.68, 4.20)	1.56 (0.51, 4.73)					
Effect of T3 within the strata of educational level	2.88 (1.24,6.66)*	2.35(0.85,6.46)					

Note:[a] The RERI, AP, SI and Multiplicative scale was adjusted for the age, ethnicity, pre-pregnancy BMI, employment, monthly household income, health insurance status, history of adverse pregnancy outcomes, gravidity, parity, baseline serum iron and vomiting. First-trimester E-DII refers to the E-DII calculated from both dietary and supplemental nutrient intakes, thereby obviating the need for separate adjustment of individual supplement use.*: $p < 0.05$; **: $p < 0.01$; ***: $p < 0.001$.

Supplementary Table S3. Sensitivity analysis of the E-DII and educational level interaction on anemia risk using alternative binary categorizations of E-DII (T3 vs. T1+T2).

Variable	Educational level			RERI [a]	AP [a]	SI [a]	Multiplicative scale [a]
	Bachelor's degree and above	Below bachelor's degree	Effect of below bachelor's degree within the strata of E-DII				
First-trimester E-DII（groups)				2.43 (-1.43, 9.00)	0.38 (-0.39, 0.67)	1.83 (0.70, 4.79)	1.06 (0.38, 2.98)
T1+T2（< 0.81）	1(Ref.)	2.78 (1.40, 5.55)**	2.78 (1.40, 5.55)**				
T3（≥ 0.81）	2.15 (1.12, 4.10)*	6.36 (2.96, 13.65)***	2.96 (1.35, 6.53)**				
Effect of T3 within the strata of educational level	2.15 (1.12, 4.10)*	2.28 (1.04, 5.00)*					

Note:[a] The RERI, AP, SI and Multiplicative scale was adjusted for the age, ethnicity, pre-pregnancy BMI, employment, monthly household income, health insurance status, history of adverse pregnancy outcomes, gravidity, parity, baseline serum iron and vomiting. First-trimester E-DII refers to the E-DII calculated from both dietary and supplemental nutrient intakes, thereby obviating the need for separate adjustment of individual supplement use.*: $p < 0.05$; **: $p < 0.01$; ***: $p < 0.001$.

Supplementary Table S4. Association between first-trimester E-DII and anemia during the second and third trimesters (OR with 95% CI from logistic regression and RR with 95% CI from modified Poisson regression).

Variable	Model 1		Model 2	
	OR (95%CI)	RR (95%CI)	OR (95%CI)	RR (95%CI)
First-trimester E-DII score	1.30(1.13,1.51)***	1.25(1.11,1.41)***	1.34(1.15,1.57)***	1.27(1.13,1.42)***
First-trimester E-DII (groups)				
T1 (≤ -0.68)	1(Ref.)	1(Ref.)	1(Ref.)	1(Ref.)
T2 (-0.68~0.81)	1.55(0.81,2.98)	1.48(0.83,2.65)	1.55(0.79,3.07)	1.45(0.80,2.66)
T3 (≥ 0.81)	2.74(1.49,5.03)**	2.37(1.39,4.02)**	2.79(1.49,5.25)**	2.30(1.36,3.90)**

Note: Model 1 represents the basic model without adjustment for covariates. Model 2 was adjusted for the age, ethnicity, pre-pregnancy BMI, educational level, employment, monthly household income, health insurance status, history of adverse pregnancy outcomes, gravidity, parity, baseline serum iron and vomiting. First-trimester E-DII refers to the E-DII calculated from both dietary and supplemental nutrient intakes, thereby obviating the need for separate adjustment of individual supplement use. *: $p < 0.05$; **: $p < 0.01$; ***: $p < 0.001$.

Supplementary Table S5. Association between first-trimester E-DII excluding supplements and anemia during the second and third trimesters, in the overall population and in specific subgroups of non-supplement users (RR and 95% CI).

Study population	E-DII excluding supplements measure	No. of participants (Cases/Total)	Model 1		Model 2	
			RR (95%CI)	*p* Value	RR (95%CI)	*p* Value

Full population	Continuous	82/562	1.10(0.96,1.25)	0.167	1.12(0.98,1.28)	0.096
	T1 (≤ -0.64)	23/188	1(Ref.)	-	1(Ref.)	-
	T2 (-0.64~0.87)	29/187	1.27(0.76,2.11)	0.361	1.32(0.79,2.21)	0.291
	T3 (≥ 0.87)	30/187	1.31(0.79,2.17)	0.292	1.34(0.80,2.25)	0.265
Non-iron supplement users	Continuous	54/330	1.06(0.90,1.23)	0.497	1.06(0.91,1.24)	0.484
	T1 (≤-0.63)	16/110	1(Ref.)	-	1(Ref.)	-
	T2 (-0.63~0.89)	19/110	1.19(0.65,2,19)	0.581	1.14(0.64,2.04)	0.660
	T3 (≥0.89)	19/110	1.19(0.65,2,19)	0.581	1.13(0.63,2.05)	0.683
Non-vitamin B12 supplement users	Continuous	59/357	1.11(0.96,1.29)	0.157	1.13(0.97,1.31)	0.115
	T1 (≤-0.69)	16/119	1(Ref.)	-	1(Ref.)	-
	T2 (-0.69~0.80)	21/119	1.31(0.72,2.39)	0.373	1.35(0.74,2.46)	0.336
	T3 (≥0.80)	22/119	1.38(0.76,2.49)	0.292	1.38(0.75,2.53)	0.302

Note: Model 1 represents the basic model without adjustment for covariates. Model 2 was adjusted for age, ethnicity, pre-pregnancy BMI, educational level, employment, monthly household income, health insurance status, history of adverse pregnancy outcomes, gravidity, parity, baseline serum iron, vomiting, and was further adjusted for supplement use (iron, folic acid, and vitamin B12 in the full population; folic acid and vitamin B12 in non-iron users; folic acid and iron in non-vitamin B12 users).

Supplementary Table S6. Association between educational level and anemia during the second and third trimesters (OR with 95% CI from logistic regression and RR with 95% CI from modified Poisson regression).

Variable	Model 1		Model 2	
	OR (95%CI)	RR (95%CI)	OR (95%CI)	RR (95%CI)
Educational level				
Bachelor’s degree and above	1(Ref.)	1(Ref.)	1(Ref.)	1(Ref.)
Below bachelor's degree	2.40(1.49,3.88)***	2.08(1.40,3.08)***	2.82(1.66,4.80)***	2.27(1.52,3.39)***

Note: Model 1 represents the basic model without adjustment for covariates. Model 2 was adjusted for the age, ethnicity, pre-pregnancy BMI, employment, monthly household income, health insurance status, history of adverse pregnancy outcomes, gravidity, parity, baseline serum iron, vomiting and first-trimester E-DII (groups). First-trimester E-DII refers to the E-DII calculated from both dietary and supplemental nutrient intakes, thereby obviating the need for separate adjustment of individual supplement use. *: $p < 0.05$; **: $p < 0.01$; ***: $p < 0.001$.
